# Tumor retractor: a simple and novel instrument for brain tumor surgery

**DOI:** 10.1186/s12957-020-1800-8

**Published:** 2020-02-13

**Authors:** Jaejoon Lim, Kyoung Su Sung, So Jung Hwang, Duk-Hee Chun, Kyung Gi Cho

**Affiliations:** 1 Department of Neurosurgery, Bundang CHA Medical Center, CHA University College of Medicine, Seongnam, Republic of Korea; 2 Department of Neurosurgery, Dong-A University Hospital, Dong-A University College of Medicine, Busan, Republic of Korea; 3 Department of Anesthesiology and Pain Medicine, Bundang CHA Medical Center, CHA University College of Medicine, Seongnam, Republic of Korea

**Keywords:** Brain tumor, Instrument, Retraction, Solid tumor, Surgery

## Abstract

**Background:**

It is important to secure a surgical space during brain tumor surgery. One of the commonly used methods is to retract the brain. We hypothesized that the tumor can be retracted and that the normal brain tissue retraction can be minimized during surgery, and thus, the degree of collateral damage caused by brain retraction would be reduced.

**Methods:**

The tumor retractor had a 90°, hard, and sharp tip for fixation of the tumor. The distal part of the retractor has a malleable and thin blade structure. By adjusting the angle of the distal malleable part of the tumor retractor, the operator can make the retracting angle additionally. Retractors with thin blade can be used in a conventional self-retraction system. To pull and hold the tumor constantly, the tumor retractor is held by a self-retraction system. The surgical technique using a tumor retractor is as follows: The first step is to fix the retractor to the tumor. The second step is to pull the retractor in the operator’s desired direction by applying force. After the tumor is pulled by adjusting the degree of force and angle, the surgical arm should be held in place to maintain the tumor retracted state.

**Results:**

The tumor retractor was used to minimize the brain retraction, pulling the tumor in the opposite direction from the surrounding brain tissue. In clinical cases, we can apply the tumor retractor with good surgical outcomes.

**Conclusions:**

A tumor retractor can be used to pull a tumor and minimize the brain retraction.

## Background

In tumor surgery, which is often performed adjacent to the surrounding normal tissue, it is very important to provide space for effective surgical resection. For decades, several studies have already been conducted to evaluate the effectiveness and safety of retraction using a new instrument and surgical approach [1–3]. One of the commonly used methods in brain tumor surgery is brain retraction. Unlike other visceral organs, the brain tissue can be easily damaged by retraction [3–5]. Prolonged retraction of brain tissues may cause irreversible damages such as ischemia and cerebral infarction [4–6]. Therefore, when the brain retraction is required, brain damage should be minimized [7, 8].

## Methods

### Tumor retractor

**Fig. 1:**
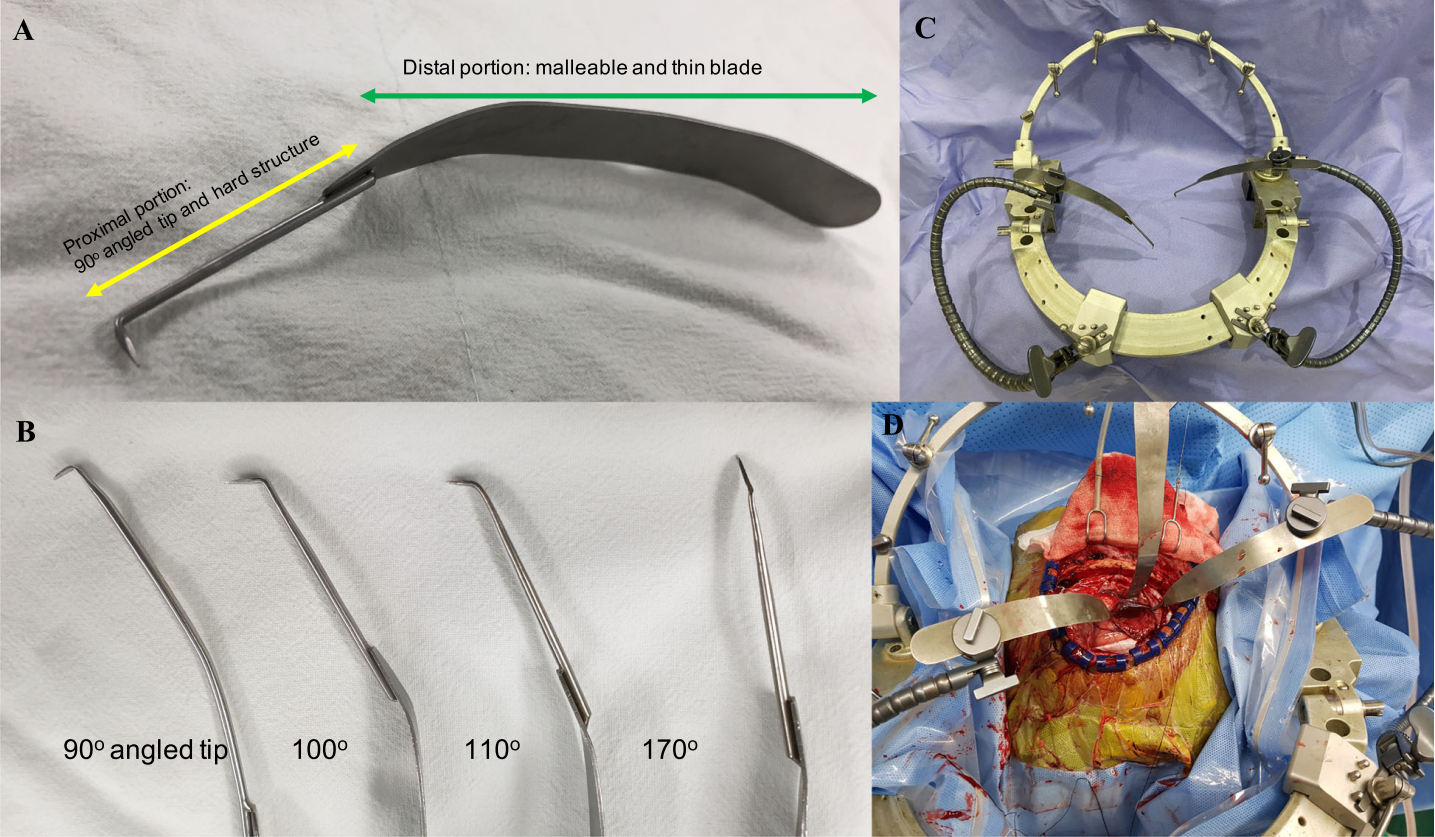
Tumor retractor. **a** Proximal portion: 90° angled tip and hard structure, distal portion: malleable and thin blade. **b** Various angled tumor retractor (90°, 110°, 120°, and 170°). **c** Applied to self-retraction system. **d** Surgical picture

Tumor retractors have a 90°, hard, and sharp tip, which is used for fixation of the tumor (Fig. 1a). The retractor tip has several angles, which are effective for pulling the tumor (Fig. 1b). The strength of the distal portion of the tumor retractor is similar to that of the malleable retractor, to provide additional angle adjustment (Fig. 1a).

The distal portion of the tumor retractor resembles a general thin brain spatula for easy adaptation to the existing self-retraction system (Fig. 1c, d). To keep the tumor retractor in place to provide proper pulling force and timing, the self-retraction system was used (Fig. 1c, d).

### Surgical technique using the tumor retractor

**Fig. 2:**
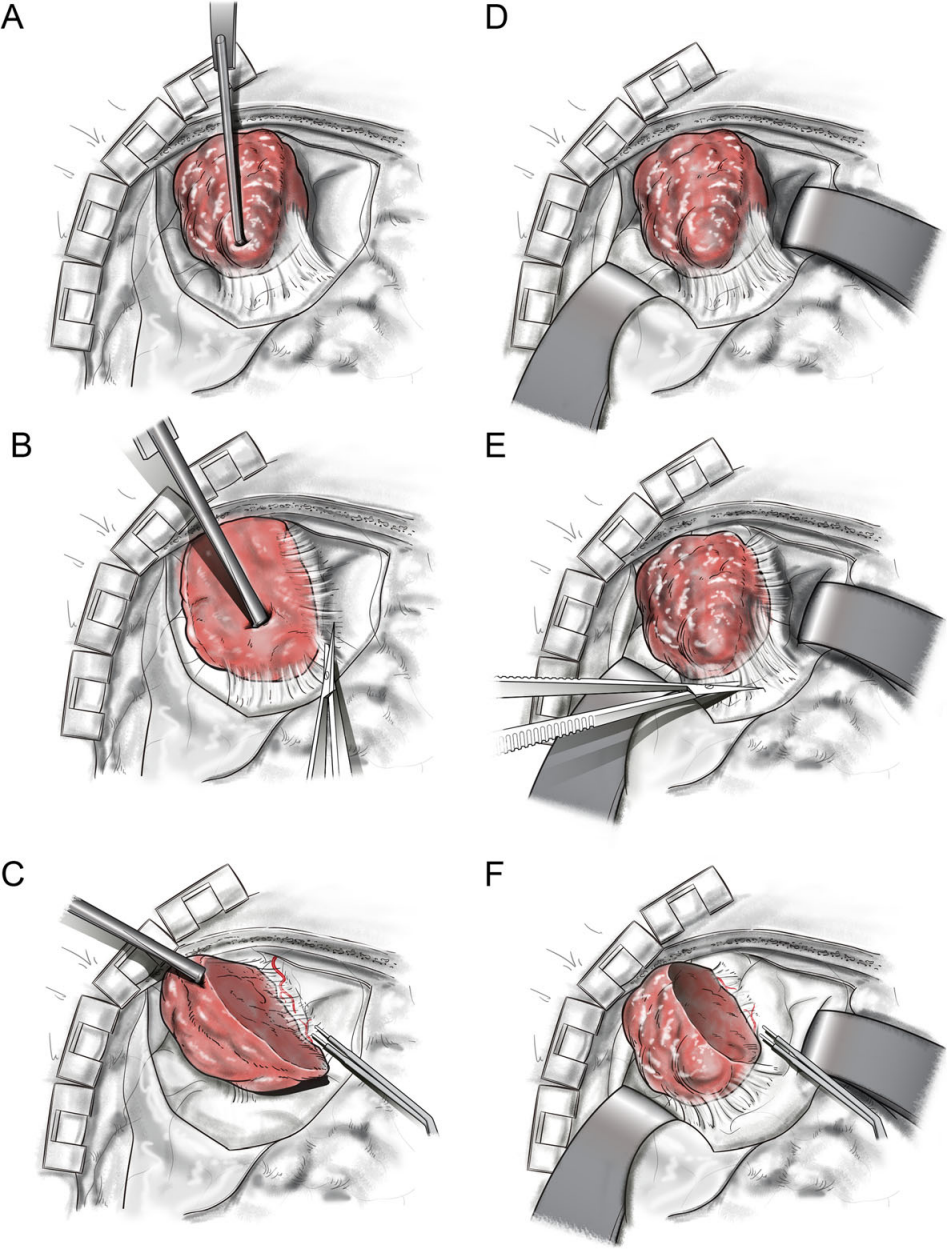
Surgery with the tumor tractor. **a** The tumor retractor was fixed to the tumor, and the retractor was pulled to the superior direction to secure the space. **b** After fixing and holding the tumor in place, the operator can perform the delicate dissection procedure. **c** If the tumor mass is removed, a wider surgical space can be secured, and it would be easier to perform the retraction in various directions. Surgery without the tumor retractor. **b** Surgical space is obtained with retraction of the surrounding brain tissue. **d** The operator can perform the delicate dissection procedure with brain retraction. **f** After the tumor was removed, a wider surgical space was secured

The tumor retractor is used in two steps (Fig. 2a).

The first step is to fix the retractor to the tumor. Depending on the consistency of the tumor, the sharp tip is firmly fixed to the tumor, to easily retract the tumor to the desired direction by applying force.

The second step is to pull the retractor in the operator’s desired direction (usually opposite to the adjacent brain) and force. After the tumor is pulled by adjusting the degree of force and angle, the surgical arm is held in place to maintain the tumor retracted state.

When the tumor is retracted following the steps described above, the operator can perform the delicate dissection procedure (Fig. 2b). The smaller or debulked the tumor mass was during the operation, the easier it is to retract the tumor in various directions and using various forces (Fig. 2c).

This study was approved by the Institutional Review Board of Bundang CHA Medical Center.

## Results

The authors have been using this tumor retractor in brain tumor surgery for more than 20 years.

A tumor retractor is easier to use in solid tumors.

### Illustrative cases

#### Case 1 (Video clip 1)

**Fig. 3:**
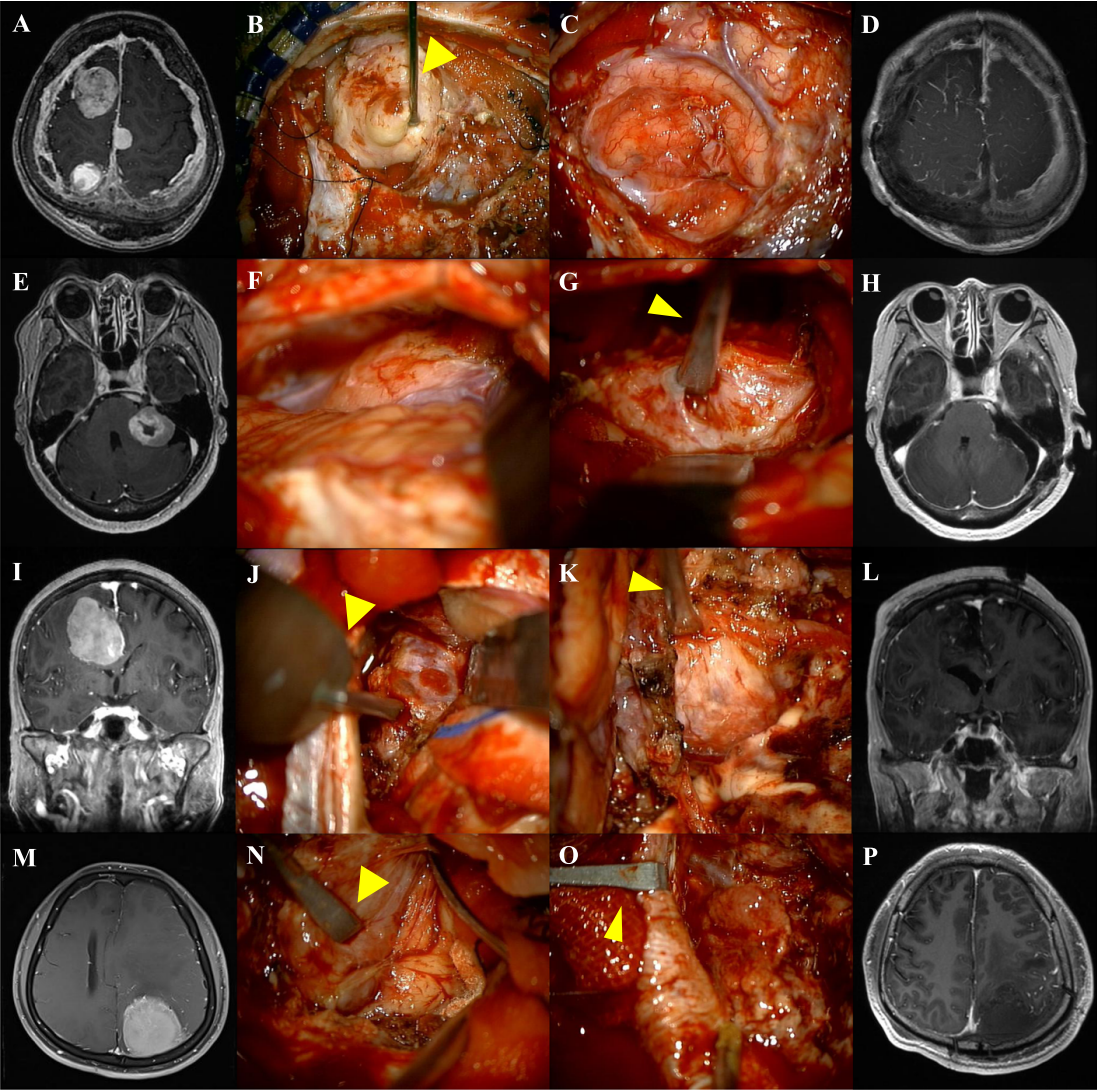
**a**, **e**, **i**, **m** Initial preoperative magnetic resonance (MR) image. **b**, **c**, **f**, **g**, **j**, **k**, **n**, **o** Intra-operative images showing the tumor retractor in place. **d**, **h**, **l**, **p** Postoperative gross total resection MRI images

A 43-year-old woman was admitted to our hospital with headache and motor weakness. She had been diagnosed with neurofibromatosis type II. She previously underwent two operations. Magnetic resonance imaging (MRI) using gadolinium contrast agent showed a well-enhanced mass in the right frontal lobe (Fig. 3a). Hence, frontal craniotomy was performed. The patient had a solid tumor attached to the surrounding tissues. The tumor retractor was fixed to the tumor, and the tumor was pulled to the superior direction to secure the space (Fig. 3b). As the operator becomes more familiar with using a tumor retractor, the procedure could be performed in one step as shown in the video clip. Dissection between the tumor and brain tissue was performed comfortably while the tumor retractor was held in place. Subsequently, we could remove a large proportion of the main mass using the tumor retractor, and the normal brain tissue retraction could be minimized. When the tumor mass was removed, a wider surgical space was secured, and it was easier to perform the retraction in various directions. Immediately after the operation, the patient did not manifest any signs of neurological deficit. Postoperative MRI showed gross total removal of the tumor (Fig. 3d).

#### Case 2 (Video clip 2)

A 69-year-old woman was admitted to our hospital with headache and hearing loss. MRI with gadolinium contrast agent showed a well-enhanced mass in the left cerebellopontine area (Fig. 3e). Surgery was performed through the lateral suboccipital approach. The patient had a solid tumor, which adhered to the surrounding brain tissues (Fig. 3f). The operator found a suitable spot to fix the retractor in the tumor and fixed it firmly. Then, the operator pulled the retractor and held it in place in order to maintain the retracted status (Fig. 3g). Subsequently, the tumor was retracted using the tumor retractor with minimal cerebellum retraction. The tumor could be dissected successfully with no adjacent brain and cranial nerve injury. Immediately after the operation, the patient did not manifest any signs of neurological deficit. Postoperative MRI showed gross total removal of the tumor (Fig. 3h).

#### Case 3 (Video clip 3)

A 46-year-old man was admitted to our hospital with severe headache and mild motor weakness. MRI with gadolinium contrast agent showed a well-enhanced mass in the left parietal area (Fig. 3m).

If the margin is clearly distinguished, such as that shown in Additional file 1: Video clip 1, the tumor can be removed relatively easily. In patients with tumor with brain invasion like this case, it is difficult to accurately differentiate the adjacent areas from the margin of tumor. After pulling the tumor using the tumor retractor, a surgical space was secured between the tumor and the surrounding tissue; then, a delicate dissection or detaching procedure was performed (Fig. 3n, o). Thus, surgery can be performed with minimal collateral damage and retraction of the normal brain. Immediately after the operation, the patient did not manifest any signs of neurological deficit. Postoperative MRI showed gross total removal of the tumor (Fig. 3p).


**Additional file 1.** Video clip 1.


## Discussion

One of the most important considerations in brain surgery is the protection of the normal brain [2, 9].

It is not easy to create an operative corridor up to the surgical target site in the brain, which has a very delicate neural structure that fills the small space called the skull. In patients with brain tumor, the tumor occupies this space. The expert should have the knowledge and skills to secure an effective surgical corridor with minimal normal brain injury.

Ongoing studies evaluate whether the surgical approach and methods used in the treatment of brain tumor can minimize the damage to the normal brain [1, 10, 11]. Gravity-based less-retraction surgery includes the interhemispheric occipital transtentorial approach and the supracerebellar infratentorial approach [12–14]. The operator could expect the retraction effect, in which the brain naturally falls due to the effect of gravity. In the lateral suboccipital approach, retractor-less surgery was performed with a large schwannoma case in the cerebellopontine angle area [15]. The authors could make the surgical space by drainage of cerebrospinal fluid and gravity-based position.

Many tools have been developed for effective and safe retraction during brain surgery. In the 1960s, a retractor called a brain elevator was designed [16]. Since then, several retractors with different sizes and shapes have been developed [17–19]. After the development of clamps to fix surgical instruments on the skull, a self-retraction system has become available [20]. The Mayfield head fixation system or Surgita system has been developed [21]. It is currently being used as a brain self-retraction system in various ways [22]. Various methods such as retraction using stitch and retraction using a pad with microbubbles have also been developed [23–25]. Recently, studies using a tubular retractor system to reduce collateral brain damage in deep seated tumor surgery have been actively performed [26–29].

Most of the existing retractors are designed to retract the brain like Leyla and malleable retractor [3, 30–32]. A tumor retractor was designed to secure the surgical space by pulling the tumor and consequently less retraction of the brain. Unlike most retractors with blunt tips, the proximal part of the retractor was designed to have a sharp tip and strong structure as shown in Fig. 1a. To fix the tumor firmly, a retractor with a 90° hard tip was required to support a sufficient pulling force. Additional angled tip retractor was designed for cases that require positioning of retractors in a certain angle (Fig. 1b). The distal part of the retractor was designed to be malleable and have a thin blade structure (Fig. 1a). By adjusting the angle of the distal malleable part of the tumor retractor, the operator can make the desired angle additionally. Retractors with thin blades can be used in a conventional self-retraction system (Fig. 1c, d). It is important to pull and hold the tumor constantly; in this study, the tumor retractor was held by a self-retraction system. Hook retractor has a sharp tip, but it is difficult to apply it to a self-retraction system and thus difficult to maintain a constant pulling force and direction [33–35].

In general brain tumor surgery, surgical space is obtained with retraction of the surrounding brain tissue (Fig. 2d–f). This instrument is primarily used to minimize brain retraction, pulling the tumor in the opposite direction from the surrounding brain tissue.

When the tumor is retracted, the adjacent arachnoid and normal brain tissues are also retracted. Therefore, tumor retraction can tear the surrounding normal tissues and vessels.

In our cases, we were able access the operation field using the tumor retractor with good surgical outcome (Fig. 3). It was easier to apply the tumor retractor when the tumor tissue was solid. Even in patients with very soft tumors, the tumor retractor could be applied by adjusting its direction and force.

### Advantage of the tumor retractor

The tumor retractor can be firmly fixed to the tumor.

The operator can pull the tumor in various direction and angle.

The existing self-retraction system is easy to apply.

Since the existing self-retraction system is used in combination with a tumor retractor, there is no need for another operator to hold the retractor in place.

### Limitation of tumor retractor

In patients with soft tumors, it is difficult to fix the tumor firmly.

Injuries can occur when the operator retracts the tumor.

### Falx retraction

Occasionally, it is necessary to retract the falx or remove the falx to remove tumors beyond the midline. One can choose to expose both sides to the surgical field. However, if it is not required to increase the length of the skin incision to expose the opposite site, the tumor can be removed with an effective falx incision or retraction. Falx is a very hard tissue that is difficult to retract with a normal retraction blade.

This retractor can be used to manage falx and adjacent sinus. For example, it is difficult to remove a tumor involving the superior sagittal sinus as bleeding may occur. Temporary sinus compression through sinus wall retraction can also aid in the removal of tumor involving the sinus and wall repair (Additional file 2: Video clip 2, Fig. 3o).


**Additional file 2.** Video clip 2.


## Conclusions

We introduced the tumor retractor that can pull a tumor and minimize the brain retraction (Additional file 3, Video clip 3).


**Additional file 3.** Video clip 3.


## Data Availability

Data sharing is not applicable to this article as no datasets were generated.

## Abbreviation

MRI: Magnetic resonance imaging
